# *Denoising Search* doubles the number of metabolite and exposome annotations in human plasma using an Orbitrap Astral mass spectrometer

**DOI:** 10.21203/rs.3.rs-4758843/v1

**Published:** 2024-07-25

**Authors:** Fanzhou Kong, Tong Shen, Yuanyue Li, Amer Bashar, Susan S. Bird, Oliver Fiehn

**Affiliations:** 1 Chemistry Department, One Shields Avenue, University of California Davis, Davis, CA, 95616, USA; 2 West Coast Metabolomics Center, University of California Davis, Davis, CA, 95616, USA; 3 Thermo Fisher Scientific, 355 River Oaks Pkwy, San Jose, CA 95134, USA

## Abstract

Chemical exposures may impact human metabolism and contribute to the etiology of neurodegenerative disorders like Alzheimer’s Disease (AD). Identifying these small metabolites involves matching experimental spectra to reference spectra in databases. However, environmental chemicals or physiologically active metabolites are usually present at low concentrations in human specimens. The presence of noise ions can significantly degrade spectral quality, leading to false negatives and reduced identification rates. In response to this challenge, the *Spectral Denoising* algorithm removes both chemical and electronic noise. *Spectral Denoising* outperformed alternative methods in benchmarking studies on 240 tested metabolites. It improved high confident compound identifications at an average 35-fold lower concentrations than previously achievable. *Spectral Denoising* proved highly robust against varying levels of both chemical and electronic noise even with >150-fold higher intensity of noise ions than true fragment ions. For human plasma samples of AD patients that were analyzed on the Orbitrap Astral mass spectrometer, *Denoising Search* detected 2.3-fold more annotated compounds compared to the Exploris 240 Orbitrap instrument, including drug metabolites, household and industrial chemicals, and pesticides. This combination of advanced instrumentation with a superior denoising algorithm opens the door for precision medicine in exposome research.

## Introduction

Human diseases are influenced by both genetic predispositions and environmental factors (GxE). Environmental impacts, including diet, lifestyle, and biological and chemical exposures, account for over 70% of disease incidence^1^. However, chemical exposures in human samples are usually low abundant at trace levels, similar to levels of physiologically active metabolites such as oxylipins, endocannabinoids or modified bile acids^2, 3^. Nontargeted exposome research as well as metabolomics and lipidomics methods rely on liquid chromatography coupled with high resolution mass spectrometry (LC-MS/MS)^4^. These small molecules are identified by matching experimental spectra against established repositories like the NIST23 library, MassBank of North America (MassBank.us), or GNPS/MassIVE^5, 6^. While the quality of the experimental spectra is critical for accurate MS/MS matching, mass spectra are often compromised by both electronic noise and chemical noise^7–9^. This problem is particularly pronounced in metabolomics and exposome studies, where the prevalence of low-abundance compounds can greatly exceed that of high-abundance compounds^2^. Electronic noise originates from the inherent characteristics of the electrical system, the discrete nature of ion signals, or the process of Fourier transformation^7, 10^. Chemical noise is derived from components in the sample that confound the signal generated by the metabolites and exposome compounds of interest^9^. Chemical noise in LC-MS/MS is defined as isobaric interferences that emerge from the testing materials themselves, or from laboratory consumables, solvents, cross-contaminations, buffers or carryovers^9^. The amalgamation of true and contaminant fragment ions produces chimeric spectra. Chimeric spectra drastically affect spectral matching scores and result in false negatives peak annotations, contributing to the ‘dark matter of metabolomics’^11, 12^. In proteomics, methods for noise removal resort to intensity modeling^13, 14^ and matching to in-silico spectra^15^. In nontargeted small-molecule studies, methods were developed that required specific experimental conditions to monitor the precursor-fragment ratios^16^, or leveraging database assistance^17, 18^. Overall, these methods provided only modest enhancements and low throughput. In addition, public datasets^19^ frequently lack the experimental settings and database metadata required for these methods. Consequently, existing denoising methods are unsuitable for large scale, standardized de-noising in metabolomics. Typical metabolomics data processing software denoises spectra by simply discarding ions below 0.5–1% of the base-peak height^20–22^. Surprisingly, the chemistry information revealed by the fragment peaks are not considered when determining if a given fragment is true ion or noise. We here show that integrating intensity modeling with assessing chemical plausibility greatly enhances the effectiveness of noise ion removal, termed *Spectral Denoising*. *Spectral Denoising* first eliminates electronic noise by stratifying fragment ion intensities, followed by filtering the remaining fragments based on their chemical plausibility as true fragments of the molecular formula of the precursor ion. Utilizing a 13-stage series dilution dataset of 240 small molecules generated an experimental benchmarking dataset with varying levels of spectral quality. This dataset was used to rigorously benchmark the robustness of *Spectral Denoising* against other methods, including by virtually adding different levels of contaminating chemical and electronic noise ions. False discovery rates (FDR) were thoroughly tested against 1,267 experimental spectra that were annotated by NIST23, MassBank.us and GNPS libraries. By integrating *Spectral Denoising* into the spectral matching process (‘*Denoising Search*’), we evaluated its performance using human plasma samples from AD patients acquired with advanced Asymmetric Track Lossless (Astral) mass spectrometry and classic Orbitrap instruments. The number of annotated compounds increased more than 2-fold with *Denoising Search* compared to classic compound annotation pipelines. By combining Astral mass spectrometry and *Denoising Search,* low-abundance exposome compounds can be detected in human plasma that have not been reported before in the literature. Hence, *Denoising Search* may herald a new era in the identification of key biomarkers, more confident compound annotations and better interpretability of datasets that are critically needed for biomedical research like neurodegenerative diseases.

## Results

**Figure 1.**
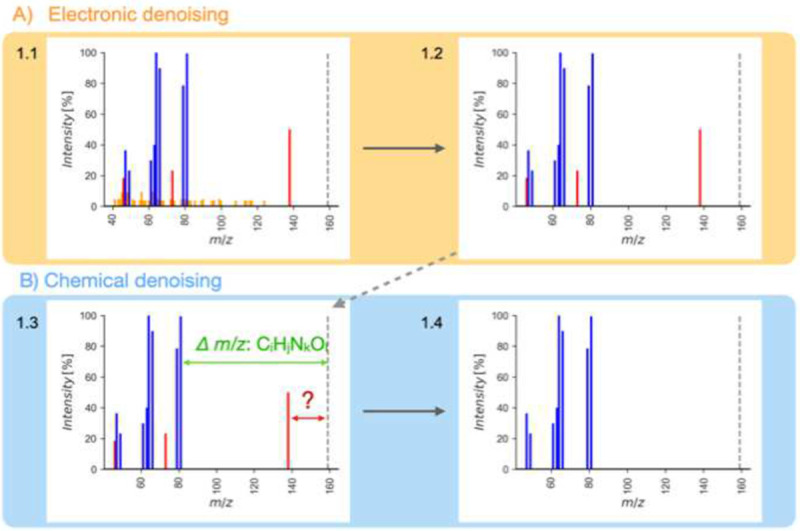
Flowchart for *Spectral Denoising*. (**a)** Removing electronic noise by recognizing repeated fragment ions with identical intensities. (**b**) Removing chemical noise by identifying remaining fragment ions that do not fit possible elemental subformulas from the precursor ion mass. The dotted line indicates the precursor ion *m/z*.

Figure 1 gives the schema of how the *Spectral Denoising* algorithm removes ions recorded in collision-induced MS/MS spectra that do not represent genuine fragments of the precursor ion. The first step removes electronic noise that commonly appears as a multitude of (low-abundant) ions with very similar intensities, also termed ‘grass noise’^23^. Chemical noise is harder to recognize because it is generated by co-isolating and fragmenting non-target precursor ions in low-resolution quadrupole mass filters that precede the collision-induced dissociation even in high-resolution mass spectrometers^24^. In practice, the isolation window used in most metabolomics studies ranges from 1 to 5 Da, increasing the likelihood of inclusion of contaminant ions due to isobaric interference^25^. These chemical noise ions can vary widely in intensity, making them difficult to distinguish from true fragment ions by manual inspection alone^9^. Hence, our schema to remove chemical noise ions is based on the unique property of true fragment ions to produce a chemically plausible neutral loss (or radical losses), calculated from the accurate mass of the target precursor ion^26^ (Figure 1).

To test this concept, we first empirically probed all 230,000 MS/MS spectra of the NIST23 library that was generated by the U.S. National Institute of Standards and Technology through a rigorous validation and fragment verification process to guarantee a high fidelity of data quality. For more than 99.5% of all ions per spectrum, fewer than four ions were found within relative intensities of ±0.1% (Extended Figure 1). This data served as valid threshold to automatically identify electronic noise ions in experimental MS/MS spectra and remove these ions, independent of the relative intensity (Figure 1A).

Electronic denoising differs in two key aspects from simply discarding ions below a predefined threshold. First, ions are not discarded simply based on relative intensity levels. In this way, electronic denoising retains low-abundant ions that may represent true fragment ions of the precursor molecule. This step is important because many small molecules do not produce fragment-rich spectra, unlike peptides, covered in the new concept of spectral entropy^21, 27^. The denoised MS/MS example spectrum in 1.4 (Figure 1) would calculate *S*= 1.9, compared to a more disordered contaminated spectrum 1.1 with *S*=3.3 before the denoising process. Second, electronic noise becomes relatively more prominent for MS/MS spectra that originated from very low-intensity precursor ions. Therefore, a simple cut-off threshold at 1% base peak intensity does not suffice for metabolomics or exposome nontargeted studies that aim at low abundant molecules^20, 22^.

Subsequently, chemical denoising identifies and removes chemical noise ions by calculating whether the exact mass of each fragment can logically be associated with a subformula loss from the parent molecular ion species (Figure 1B). The chemical plausibility of relative loss subformulas was validated using the Seven Golden Rules algorithm to discard chemically impossible losses (e.g., CH_12_)^28^ while ignoring the LEWIS and SENIOR checks that are designed for intact molecules. In collision-induced dissociation, a fragment ion can result from multiple relative losses from the precursor ion, potentially violating the SENIOR rule^29^.

Instead, chemical denoising expands the logic of our subformula-loss calculations of chemical noise ions. For example, radical fragment ions are formed in about 10% of small molecule MS/MS spectra as metastable state in mass spectrometry, even for even-electron precursors^30^. Enforcing the LEWIS rule would lead to the removal of these valid radical fragment ions^30^. Moreover, about 1% of MS/MS spectra were reported in which the collision gas nitrogen formed bonds with substituted aromatic compounds within the collision cell, with subsequent background water substitution^31, 32^. We confirmed the occurrence of such fragmentations in the NIST23 spectral library and validated these experimentally in our laboratory. Hence, *Spectral Denoising* accounts for possible [M+N_2_+H_2_O] molecule reactions when calculating the relative loss of fragment ions for substituted aromatic compounds. The sequential combination of electronic and chemical denoising was validated on 10,000 NIST23 spectra and compared MS/MS similarities of the denoised against the original library spectra (Extended Figure 2). Entropy similarity is scaled in the same manner as classic dot-score similarities, from 0–1 with 1 marking perfect matches and 0 giving no similarity at all. If no ions were removed by the denoising algorithm, MS/MS similarities would remain identical, leading to perfect matching scores of 1. In all calculations, the remaining abundance of the precursor ion intensities is ignored to focus entirely on genuine fragment spectra, and because all identity-search algorithms already exclude compounds that do not match specific accurate mass windows of the precursor mass (typically at 5 ppm). The average similarity of the selected denoised NIST23 spectra was >0.99, proving that the denoising method did not accidentally remove true ions, and that the NIST23 spectra have very high quality (Extended Figure 2).

### Denoising experimental MS/MS spectra of 240 metabolites diluted from 500–0.02 pmol injections

To evaluate the effectiveness of our denoising strategy, we analyzed 240 metabolites in both positive and negative electrospray ionization (ESI) modes in a 13-step serial dilution from 500 pmol to 0.02 pmol injected onto the column. MS/MS spectra of the most concentrated 500 pmol injections represented optimal spectral quality, while the more diluted ones were expected to gradually deteriorate in spectra quality due to an increased contribution of contaminating noise ions. A total of 28 compounds were excluded due to insufficient fragmentation with less than two fragment ions (spectral entropy <0.5). The remaining dataset of MS/MS spectra that were used for MS/MS similarity calculations included a total of 11,823 spectra, encompassing 6,885 in the positive mode and 4,938 in the negative ESI mode (Extended Figure 3).

**Figure 2.**
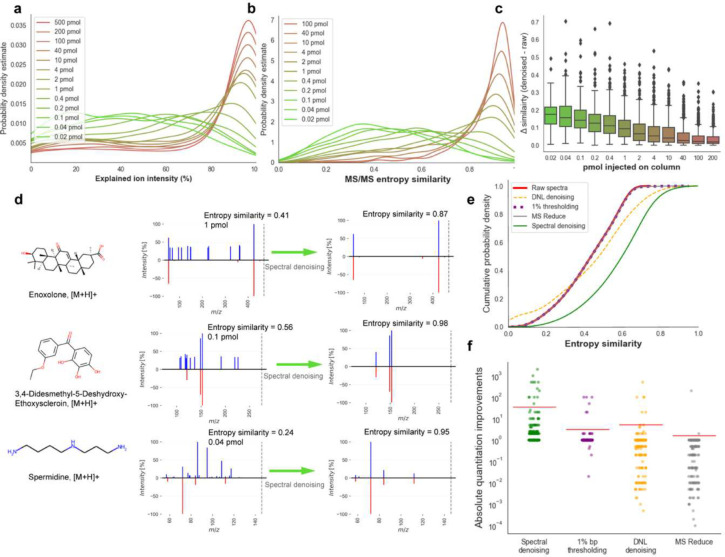
Developing, validating and benchmarking the *Spectral Denoising* algorithm. (**a)** Probability density distribution (explained denoised/raw intensities) of all MS/MS spectra from 240 injected standards between 0.02–500 pmol. **(b)** Probability density distribution of the entropy similarities before *Spectral Denoising* of all MS/MS spectra from 240 injected standards between 0.02–200 pmol, using the 500 pmol spectra as reference. **(c)** Improvement in spectral entropy similarities after *Spectral Denoising*. **(d)** Examples of head-to-tail plots of MS/MS spectra before and after *Spectral Denoising* for compounds injected at low quantities. **(e)** Cumulative distribution of MS/MS entropy similarities before (‘raw’) and after applying three benchmarking methods against the *Spectral Denoising* algorithm. Only raw spectra with MS/MS entropy similarities <0.75, a typical threshold for automatic metabolite annotations. **(f)** Strip plot to visualize absolute improvements in MS/MS similarities across three benchmarking methods and the *Spectral Denoising* algorithm.

First, the ability of the denoising algorithm was evaluated to discern noise ions from genuine fragment ions. To this end, the total explained ion intensity was enumerated as metric to quantify the proportion of true ion intensities in each spectrum (Figure 2a). The probability density of explained ion intensities shifted markedly from high to low amounts of injected compounds, highlighting the effectiveness of the denoising algorithm to identify noise ions. The probability distributions of the calculated entropy similarities (Figure 2b) showed and even more pronounced decrease in median MS/MS similarities with lowered injected amounts, from >0.92 entropy similarity at 200 pmol injected to <0.41 median similarity at the lowest injection quantities. Remarkably, at 1 pmol injections (about 0.3 ng injected onto the column, at the median molecular mass of the chemicals included test mixture), more than 50% of all MS/MS spectra already failed to match the reference spectra at entropy similarity > 0.75, a cut-off that is often used in metabolite annotations in metabolomics (Figure 2b). More importantly, the *Spectral Denoising* algorithm effectively removed chemical and electronic noise ions for all test compounds (Figure 2c). As expected, the largest improvement for MS/MS similarity calculations were observed for very low injected amounts with a median gain of 0.18 entropy similarity scores. At 1 pmol injections, a median improvement of 0.1 MS/MS entropy similarity gain was noted, and even for 200 pmol injections, 25% of the compounds already showed an improvement of entropy similarities of 0.05 (Figure 2c). Example spectra are depicted in Figure 2d with the precursor ions given as dotted lines to indicate that residual precursor mass intensities were ignored in MS/MS similarity calculations. For 3,4-didesmethyl-5-deshydroxy-ethoxyscleroin and enoxolone, raw spectra MS/MS of low abundant injections were notably marred by substantial electronic noise with up to 30% relative base peak intensity, vastly exceeding the typical 1% base peak ratio often used as a threshold (Figure 2d). For spermidine injected at 0.04 pmol, the initial raw spectrum displayed a diverse set of ion intensities without obvious signs of electronic noise. Surprisingly, the three most abundant ions m/z 86.004, m/z 95.009, and m/z 108.494 were identified as chemical noise, compromising the entropy similarity to a level of 0.24 score. The denoised spermidine spectrum showed a perfect match with an entropy similarity of 0.95 (Figure 2d).

Next, the efficiency of *Spectral Denoising* was evaluated by benchmarking its performance against three established MS/MS denoising techniques: the classic 1% base peak height thresholding method^20, 22^, dynamic noise level (DNL) denoising^14^, and MS Reduce denoising^13^_._ The benchmarking dataset comprised all 2,677 spectra that had raw MS/MS similarities <0.75 score, meaning they might not be annotated by high confidence identification schemas (Figure 2e). Similar to *Spectral Denoising*, the three methods selected for benchmarking purpose are standalone tools with a generalized scope of applicability, as they do not necessitate complementary metadata or additional experimental setups. While we initially hypothesized that all denoising methods might enhance spectral matching, neither of the three benchmarked algorithms showed any marked improvement. DNL denoising yielded only a slight improvement in entropy similarity for a limited subset of spectra, with a modest increment of 0.01 in spectral similarity. The MS Reduce approach, even at the highest quantization level of 11^13^, failed to enhance spectral similarity effectively. Surprisingly, even the classic 1% base peak thresholding method showed negligible impact on spectral similarity matching, indicating that while simple and computationally inexpensive, this method is inadequate for noise ion removal in low-abundance compound spectra. In contrast, our *Spectral Denoising* method showed significant gains for MS/MS spectral matching with a median entropy similarity increase of 0.17, lifting more than 1,500 spectra to MS/MS similarity >0.75 and thereby boosting the compound annotation rates by 30% (Figure 2e).

A second benchmarking test quantified at how much lower injected quantities compounds could be annotated at entropy similarity >0.75 by applying the *Spectral Denoising* method. For all 181 compounds that were detected in at least more than one dilution stage (Supplement 1), the fold-change was calculated between the lowest injected amount that reached >0.75 entropy similarity for the raw MS/MS spectra, compared to the lowest injected amount after *Spectral Denoising* (Figure 2f). On average, our *Spectral Denoising* method required 35-fold lower molar quantities injected onto the column (Figure 2f, Supplement 1), while not a single compound failed to be annotated after *Spectral Denoising* (no false negatives). In contrast, all other denoising techniques showed minimal improvements for annotations injected at lower absolute quantities. Specifically, the 1% base peak thresholding method demonstrated no enhancement for 163 compounds. Similarly, DNL denoising and MS Reduce only showed improvements for so-few compounds. More concerningly, both DNL denoising and MS Reduce did not only fail to improve quantity thresholds for successful MS/MS annotations but instead detrimentally affected spectral matching. For MS Reduce, 108 compounds became unannotated (false negative) even at the same concentration level after processing the raw MS/MS spectra. For DNL denoising, this number of false negatives was 86 compounds. This indicates that both methods inadvertently removed true fragment ions, thereby shifting entropy similarity distributions to lower values across all spectra (Extended Figure 4). This disparity in performance across denoising techniques may originate from their foundational assumptions, as both DNL and MS-reduce were introduced on proteomics data that are usually fragment-rich, unlike in metabolomics where collision-induced fragments spectra are usually sparse. Thus, the underlying assumptions of the DNL- and MS-reduce intensity modeling-based denoising methods are no longer valid when applied to metabolomics data, highlighting the necessity for specialized approaches in this field.

### Applying Spectral Denoising against artificial noise ions

Contamination by noise ions in experimental spectra from biological samples is more challenging than the sets of chemical standards shown above. A larger diversity of noise origins, e.g. from the chemosphere of the exposome, requires better mimicking large-scale contribution of different types of noise. To thoroughly assess the robustness of the tested denoising algorithms, the 0.01–200 pmol dilution series experimental spectra were used to create artificial chimeric spectra by adding simulated levels of chemical and electronic noise. Both types of noise ions were introduced using established noise models^8^, albeit with different parameter sets to accurately reflect their characteristics. Chemical noise, characterized by real chemical formulas, typically appears with high intensity but low ion counts. The mass-to-charge ratios of chemical noise ions were sampled from a database of 3.5 million authentic chemical formulas^33^, with their relative intensities determined using the noise model with a mean intensity of 50% of the base peak height. We defined three contamination levels for chemical noise, with the total noise ion counts to raw ion counts in a ratio of 1:10 (low), 2:10 (medium), and 5:10 (high). Electronic noise typically manifests as low-intensity but high-quantity ‘grass noise’^23^. Thus, the mass-to-charge ratios of electronic noise ions were randomly sampled, with their relative intensities determined by the same noise model mean ion intensity of 5% base peak height. Similarly, we also designed three levels of electronic noise contamination: low (2:1), medium (10:1), and high (100:1), yielding nine tests of combined noise levels.

**Figure 3.**
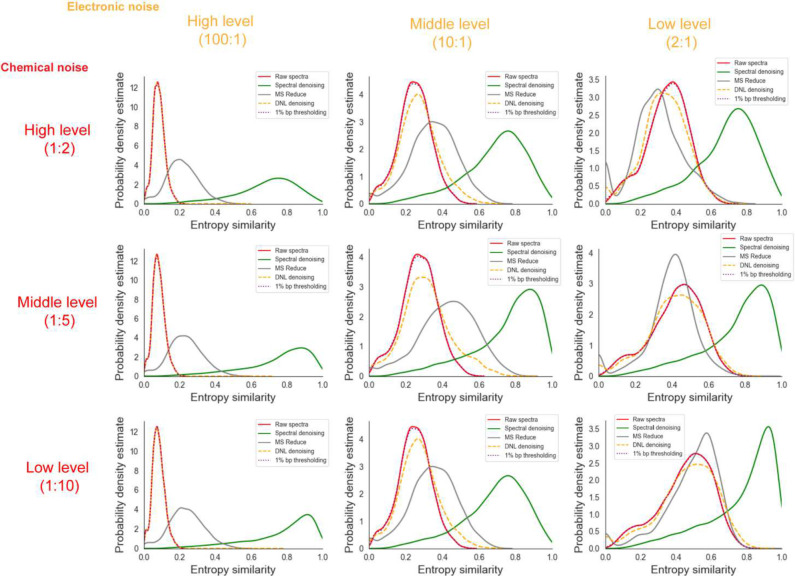
Probability distributions of MS/MS entropy similarities before (‘raw’) and after applying three benchmarking methods against the *Spectral Denoising* algorithm, under varying levels of artificially added chemical and electronic noises. Chemical noise: *Level 1:10* added at least one noise ion for every 10 experimental ions; *Level 1:5* added at least one noise ion for every 5 experimental ions; *Level 1:2* added at least one noise ion for every 2 experimental ions. Electronic noise: *Level 2:1* added two noise ions per experimental ion; *Level 10:1* added ten noise ions per experimental ion; *Level 100:1* added one hundred noise ions per experimental ion.

The resulting chimeric spectra were matched against the 500 pmol benchmark spectra of the authentic standards, and denoised with *Spectral Denoising*, MS Reduce, DNL denoising and 1% bp thresholding (Figure 3). The distribution frequency plot of all combined raw spectra of the compound dilution series yielded a median entropy similarity of 0.8, with an average of 0.71 and a mode at 0.95 (Extended Figure 4). Adding virtual noise to render chimeric spectra drastically reduced the spectral quality in all nine test scenarios, even for the lowest level of chemical and electronic noise (Figure 3), to a mode of 0.5 spectral entropy. When considering the mode points in the frequency distributions of the raw spectra, electronic noise worsened spectral entropy scores more dramatically than chemical noise additions, even at low levels of electronic noise. For all nine test cases, our denoising method restored the frequency distributions of the contaminated spectra above the levels of the original spectra, with the median spectral entropy similarity ranging from 0.71 (high electronic, high chemical noise) to 0.87 (low electronic, low chemical noise) (Figure 3). Importantly, the benchmarking test clearly demonstrated that none of the other algorithms came close to the performance of our *Spectral Denoising* method (Figure 3), with the best frequency modes located at spectral entropy 0.6 for the MS Reduce method for the low electronic, low chemical noise test scenario.

**Figure 4.**
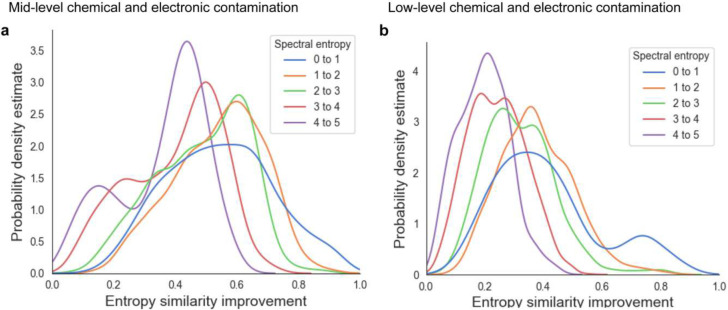
Density distributions for MS/MS similarity improvements after *Spectral Denoising* for all MS/MS spectra from 240 injected standards between 0.02–200 pmol, using the 500 pmol spectra as reference. Chemical standards were grouped into five sets with different starting spectral entropies (blue to purple). (**a)** MS/MS similarity improvements for spectra to which contamination ions were artificially added at ‘*mid-levels*’, Level 10:1 electronic noise, and Level 1:5 chemical noise. **b** MS/MS similarity improvements for spectra to which contamination ions were artificially added at ‘*low levels*’, Level 2:1 electronic noise and Level 1:10 chemical noise.

Overall, neither the 1% bp thresholding nor the DNL denoising approaches yielded any substantial improvement across any level of chemical noise contamination (Figure 3). Last, we investigated if the improvement of MS/MS similarities by *Spectral Denoising* depended on the entropy of the 500 pmol reference spectra themselves. Spectra were categorized into five groups, from 0–1 entropy (low number of fragment ions) to 4–5 entropy levels (high number of fragment ions with varying intensities). We suspected that most experimental spectra from biological samples would only be subjected to minor to moderate contamination and therefore only used mid-level and low-level combinations of virtually added noise ions. As expected, reference raw spectra that started with lower entropy (0–1) benefitted the most from *Spectral Denoising*, as such spectra also see the most dramatic decline in MS/MS spectral similarities when noise ions are added (Figure 4). Conversely, reference raw spectra with high spectral entropy (S >4) better tolerate the addition of noise ions, and hence benefit a little less from *Spectral Denoising* (Figure 4). Yet, mass spectra from any starting entropy levels showed clear improvements in MS/MS similarity from *Spectral Denoising* when artificial noise was added, with a frequency distribution mode of 0.42 similarity improvements for S=4–5 spectra and middle levels of added noise, and 0.2 similarity improvements at low levels of added noise (Figure 4). The results for the remaining seven sets of entropy similarity improvements are given in Extended Figure 5. Overall, these sets of benchmarking and noise-addition experiments clearly demonstrates that our *Spectral Denoising* method outperforms all other techniques and is extremely robust across varying levels of chemical and electronic noise contamination.

### Development and applying Denoising Search on HILIC-MS/MS data from the plasma of AD patients

In the prior experiments, all test spectra had a priori knowledge of the molecular formula information. Although today’s algorithms are capable of annotating molecular formulas from MS/MS spectra of reference compounds with >95% confidence^33, 34^, these tests have never been conducted on low abundant or noisy spectra. To enhance the applicability of our denoising method, *Spectral Denoising* was integrated into the spectra searching process, now termed *‘Denoising Search*.’

*Denoising Search* starts by denoising the experimental spectra using all molecular formulas that fall within the predefined precursor mass accuracy, i.e. not assuming a single starting formula. As it is a spectral identity search algorithm, it depends on the formula space that is being searched. In combination, MassBank.us, GNPS and NIST23 contain 2,028,556 experimental spectra, corresponding to 435,698 compounds and 37,493 formulas. When restricting the search space in this way, spectral matching scores for the *Denoising Search* were calculated based on all denoised spectra that fit the formula criteria within the mass accuracy of the instrument. For practical reasons, a 10 mDa mass accuracy threshold was used although Orbitrap instruments are known to yield much better exact masses (i.e., sub-ppm with internal calibration). However, for low abundant ions, mass accuracy levels suffer in concordance with compromised ion statistics. Essentially, *Denoising Search* functions similarly to a Bayesian probability approach, evaluating how likely it is to observe the query spectra with all potential chemical and electronic noise removed, given a specific target compound. The rationale is that if the correct molecular information is used to denoise the spectra, noise ions will be accurately identified and removed, thereby improving spectral matching scores. Conversely, if the molecular formula information is incorrect, the fragment patterns will be vastly different, and the removal of true ions would lower the entropy similarity scores, still resulting in true negative annotations. This rationale was validated by testing the false discovery rate (FDR) *Denoising Search* against simple entropy similarity identity search on an in-house validation dataset with extensive manual curation (Extended Figure 6). At an entropy similarity level of 0.75, the two methods showed <1% differences in terms of the FDR rate, indicating that *Denoising Search* did not introduce unwanted bias or false positives (Extended Figure 6).

**Figure 5.**
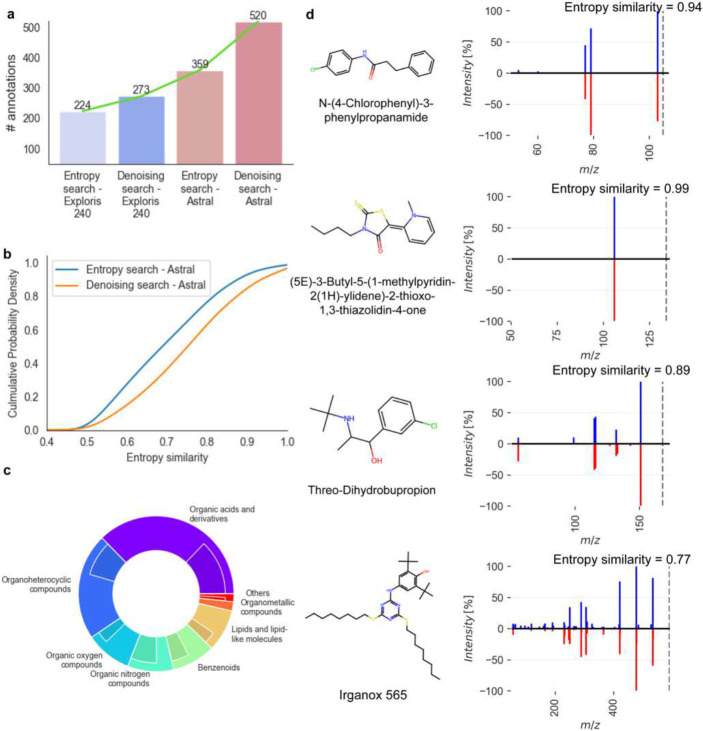
*Denoising Search* results for positive ESI mode HILIC-MS/MS data acquired on an Exploris 240 Orbitrap instrument and the Astral mass spectrometer, using 20 plasma samples of Alzheimer’s disease patients. **(a)** Improvement of metabolite annotations at MS/MS similarity >0.75 before and after *Denoising Search* using MassBank.us, GNPS and NIST23 libraries. **(b)** Cumulative probability density before and after *Denoising Search* for Astral mass spectrometry spectra. **(c)** Proportions of metabolite annotations after *Denoising Search* at MS/MS similarity >0.75 for different chemical superclasses using the Exploris 240 Orbitrap (inner ring) or the Astral mass spectrometer (outer ring). **(d)** Head-to-tail plots for four selected compounds annotated uniquely on Astral data after *Denoising Search* (blue) against library spectra (red).

To evaluate the performance of *Denoising Search* on experimental spectra of human patient samples, a small pilot plasma study was used comparing two high-resolution, accurate mass instruments: the classic Orbitrap Exploris 240 mass spectrometer and a new instrument introduced in 2023, the Orbitrap Asymmetric Track Lossless (Astral) mass spectrometer. The Astral mass analyzer acquired 17 times more spectra in each scan cycle compared to the Exploris 240 instrument, resulting in 5-times more MS1 m/z-retention time features that had corresponding MS/MS spectra. In effect, the Astral instrument provided a top-35 data dependent analysis MS/MS survey, surpassing the Exploris 240 instrument that only used a top-2 DDA mode. While the Orbitrap Astral instrument had previously shown its superior capabilities in proteomics studies, this comparison demonstrates its advantages for metabolomic tests. Overall, the raw spectra from the Astral analyzer achieved 60% more annotations than those from the Exploris 240 mass spectrometer when matching spectra against the NIST23, MassBank.us and GNPS libraries (Figure 5a). For the Exploris 240 instrument, *Denoising Search* facilitated an additional 22% increase in annotations, while Denoising Search yielded a 45% increase in annotated compounds over the raw spectra for the Astral mass analyzer (Figure 5a). Hence, compared to the raw Exploris MS/MS spectra, the *Denoising Search* on Astral data led to 2.3-fold more annotations, including many exposome compounds that were not found on the Exploris Orbitrap instrument. Notably, using *Denoising Search*, a significant increase of 0.11 median MS/MS entropy similarity was achieved for Astral spectra that showed raw entropy similarity ≥ 0.4 (Figure 5b). A closer examination of the seven main ClassyFire compound superclasses revealed a notable increase in the number of annotations across all superclasses on denoised Astral spectra compared to those found with Exploris (Figure 5c). Superclasses such as organic acids and derivatives fully leveraged the capabilities of the Astral, resulting in a 2.2-fold increase in annotated compounds, while organoheterocyclic compounds saw an 89% increase in annotated compounds. A significant increase in the number of MS/MS spectra was acquired by Astral Orbitrap mass spectrometry, thanks to its high sensitivity and its unprecedented acquisition speed (up to 200 Hz in DIA mode, 160 Hz in DDA mode). By combining our Denoising Search with Astral mass spectrometry, several compounds were identified that were previously underexplored in human blood (Figure 5d). Beyond drugs like threo-dihydrobupropion and N-(4-chlorophenyl)-3-phenylpropanamide, Irganox 565, a hindered phenol antioxidant, was reported in human blood for the first time, despite its prior detection in environmental dust samples^35^. This pilot study demonstrates that the advancement of mass analyzers allows for the acquisition of more spectra, and the *Denoising Search* is crucial for fully taking advantage of these extra spectra triggered by precursors across a wide range of magnitudes.

## Discussion

The impact of noise ions in spectra of low-abundance compounds is well-recognized in metabolomics and exposome research. These ions complicate chemical annotations, contributing significantly to the accumulation of ‘dark matter’ in small-molecule research. We here employed a strategy combining intensity modeling and subformula assignments to effectively eliminate noise ions while preserving essential true fragment ions, even at low relative intensities. Using a 13-stage dilution of MS/MS spectra of genuine chemical reference standards as a ground truth dataset, demonstrated a superior ability for the *Spectral Denoising* algorithm to identify and remove noise ions. Noise removal notably enhanced MS/MS entropy similarities, particularly for spectra that were injected at low absolute quantities. Low abundant peaks represent the large majority of unknown compounds in metabolomic studies, rendering *Denoising Search* as a promising tool for major improvements in metabolome and exposome coverage.

We benchmarked our method against three alternative denoising algorithms. *Spectral Denoising* consistently outperformed the benchmarked alternatives, improving both the entropy similarity and the absolute quantity limit of high-confidence compound annotations. Despite varying levels of artificial noise, our method maintained robust performance. However, not all added noise ions were removed, primarily because our chemical plausibility checks were limited to the algorithms embedded in the Seven Golden Rules method^28^, without considering molecular connectivity. Alternative approaches for recognizing true fragment ions involve the application of substructure annotation tools. Current software, such as Sirius^34^, and MS-FINDER^36^, often fails to recognize radical losses, which are prevalent in small molecule spectra (affecting over 60% of even-electron precursors spectra in NIST20)^30^. Without recognizing radical losses, true fragment ions may potentially be discarded. Therefore, subformula assignments that preserve valuable true fragment ions should be preferred over substructure annotation tools.

Yet, inherent limitations persist when solely relying on MS/MS spectra for compound annotation, even after removing noise ions. Reference spectra with inherently low spectral entropy are particularly vulnerable to noise ions, potentially leading to false negatives. The inability to differentiate between isomeric compounds and in-source fragments also presents significant challenges for the annotation of compounds in metabolomics when solely relying on MS/MS spectral matching. These observations indicate that employing hard thresholding based exclusively on spectral similarity is suboptimal for compound annotation in metabolomics and exposome research. Instead, *Denoising Search* should be supplemented with orthogonal experimental measures, such as retention time matching^37^, molecular cross-section comparisons^38^, and biological metadata screening^39^, to further enhance the confidence of compound identifications in small molecule research. When applied to the latest ThermoFisher Scientific instrument, the Astral mass spectrometer, *Denoising Search* facilitated a 2.3-fold increase in the number of annotated compounds compared to classic MS/MS similarity investigations on an Exploris 240 mass spectrometer, with improvements noted across all seven major chemical superclasses.

## Methods

### Spectral Denoising

High-resolution mass spectra utilized for the development and validation of our *Spectral Denoising* algorithm were sourced from the licensed NIST23 Tandem Mass Spectral Library (2023 release). The explained ion intensity was calculated as the ratio of ion intensity retained post-denoising, denoted as, $I_{p,valid}$, to the total ion intensity of raw spectra, $I_{p}$, as demonstrated in equation (1):

(1) $$Explained\;intensity\;\text{(\%)}=\frac{\sum_{p,valid}I_{p,valid}}{\sum_{p}I_{p}}$$

Figure 1 (main text) visualizes the schema of the *Spectral Denoising* pipeline. All spectra were subjected to precursor removal before applying any form of *Spectral Denoising*, to ensure that residual intensities of the precursor ions do not inflate MS/MS matching scores.

### Acquiring serial dilution MS/MS data of reference compounds to validate *Spectral Denoising*

Stock solutions of all target chemicals were prepared at 10 mM concentrations in methanol. Six mixtures of non-isobaric standards, each containing 40 compounds, were prepared by mixing 2.27 μL of each standard to achieve a concentration of 0.25 mM. 13 dilutions from these stock solutions were made to obtain final amounts to be injected into the LC-MS/MS systems, ranging from 0.02 pmol to 500 pmol: 0.02, 0.04, 0.1, 0.2, 0.4, 1, 2, 4, 10, 40, 100, 200 and 500 pmol (the concentration of the stock solution). Prior to injections, solutions were dried and resuspended in 100 ul of the LC starting buffer. 2 ul volumes were injected onto a 10 cm, 2.1 mm i.d., 1.7 um particle Waters Acquity BEH amide column maintained at 30 °C with a flow rate of 0.4 mL/min, utilizing a gradient of mobile phases of water with 0.1% formic acid (A) and acetonitrile with 0.1% formic acid (B)^40^. Mass spectrometric detection was carried out on a Thermo Q-Exactive HF Orbitrap instrument (ThermoFisher Scientific, San Jose, CA) operated in positive electrospray ionization mode. Mass spectrometry was performed from a mass range 60–1500 m/z with a sheath gas flow rate 60, auxiliary gas flow rate 25, sweep gas flow rate 2, spray voltage 3.6 kV, capillary temperature 300 °C, S-lens RF level 50, and an auxiliary gas heater temperature 370 °C. MS^1^ settings were set at a resolving power R=70,000, an automatic gain control target of 1e6, and a maximum injection time of 100 ms for single scans in centroid mode. MS/MS data were acquired in data-dependent mode at a resolving power R=15,000, an AGC target of 1e4, and a maximum injection time of 100 ms, with an isolation window of 1.0 m/z and no offset. The Top-N setting was 4, with an MSX count 1, loop count 4, and normalized collision energy steps 25, 35, and 65 NCE in centroid mode. For each mixture, precursor ions of the target compounds were specifically included for MS/MS acquisition as separate target inclusion lists to ensure that MS/MS spectra were acquired even for very low injected amounts, for which MS1 ion intensities may not have been found within the top-4 most abundant ions in an MS1 spectrum. Feature detection was performed on MS 4.9.2.

### Benchmarking Spectral Denoising against alternative denoising algorithms

Algorithms were obtained from literature based on their premise to remove noise ions in MS/MS spectra and to promote spectral annotations. Methods were excluded if they relied on data integration across multiple spectra (‘consensus spectra’) or if they required auxiliary instrumentations^16, 17^. Three alternative denoising algorithms were implemented in Python 3.8. The reducing factor used for MS Reduce denoising was 90 with the maximum allowed quantization level of 11. For threshold denoising, the widely used 1% base peak height was selected as the predefined noise level. DNL denoising algorithm does not require additional parameter settings. The performance of the denoising algorithms was benchmarked on the 240 metabolite standards with absolute injected volumes from 200 pmol to 0.02 pmol, using the 500 pmol spectra as reference spectra. The improvements of MS/MS similarities for low abundant compounds were calculated using the ratio of the lowest injected quantity of compounds that yielded an MS/MS entropy similarity >0.75 of the raw spectra, divided by the lowest injected quantity of compounds that yielded an MS/MS entropy similarity >0.75 of the MS/MS spectra after the use of the benchmarked algorithms. A ratio less than 1 indicates that spectra gave <0.75 MS/MS similarity after denoising, even for the injected quantities of compounds for which raw spectra were annotated at MS/MS entropy similarity >0.75. Spectral entropy and entropy similarity were calculated as published before^21, 27^.

### Adding chemical and electronic noise ions to MS/MS spectra

To test the robustness of the *Spectral Denoising* method against three benchmarking algorithms, chemical noise and electronic noise were artificially added to all MS/MS spectra of the 240-compound mixtures with absolute injected volumes from 200 pmol to 0.02 pmol. The relative intensity of both electronic and chemical spectral noise I was generated using a Poisson distribution, demonstrated in equation (2):

(2) $$f(I)=\frac{\lambda^{I}e^{-\lambda }}{I!}$$

Here, f(I) represents the probability that a peak with relative intensity I will be generated, where λ=50 characterizes the chemical noise and λ=5 represents the electronic noise, to accurately mimic their respective behaviors. For chemical noise, *m/z* values were randomly selected from a database of 3.5 million formulas to ensure that only chemically feasible element ratios were used, with the additional constraint that noise ions did not exceed the precursor m/z. Electronic noise *m/z* values were randomly sampled from a uniform distribution ranging from 0 to the precursor ion m/z. If the calculated number of noise ions was not an integer, it was rounded up to the nearest integer to ensure that at least one noise ion was generated for each spectrum. The improvement in MS/MS entropy similarity scores was defined as the difference in entropy similarity between the 500 pmol reference spectra and the contaminated raw spectra or the 500 pmol reference spectra and the denoised spectra. The spectral entropy was calculated based on the 500 pmol reference spectra as the ground truth.

### Acquiring and annotating HILIC-MS/MS data from plasma of AD patients using Orbitrap Exploris 240 and Orbitrap Astral mass analyzers with *Denoising Search*

The dataset comprised a subset of 20 plasma samples from an Alzheimer’s patients exposome cohort, as part of an exploratory study coordinated by Duke University under Prof. Rima Kaddurah-Daouk. Samples underwent analysis using both the Orbitrap Exploris 240 and Orbitrap Astral systems (Thermo Scientific, San Jose). The same hydrophilic interaction liquid chromatography (HILIC) method was employed for both systems, employing a Waters ACQUITY Premier BEH Amide Column (1.7 μm, 2.1 mm × 50 mm). Gradient elution used a biphasic system consisting of (a) water and (B) 95% acetonitrile, both buffered with 10 mM ammonium formate and 0.125% formic acid. The gradient started at 100% phase B, reducing to 30% over 2.05 minutes, followed by an equilibration period back to 100% B over 0.65 minutes at 0.8 ml/min. For mass spectrometry, the Exploris 240 Orbitrap was set to perform an MS1 full scan (60–900 m/z range, 60,000 resolution, 1e6 AGC target, maximum injection time 100 ms) and a top-2 data-dependent MS/MS acquisition (DDA) (15,000 resolution, 1e5 AGC target, maximum injection time 10 ms, isolation window 1 m/z, normalized collision energies of 30-50-80%). The Astral system similarly conducted full scan MS1 (60–900 m/z range, 60,000 resolution, 1e6 AGC target, maximum injection time 100 ms) and MS/MS scans (15,000 resolution, 1% of 1e5 AGC target, maximum injection time 10 ms, isolation window 1 m/z, normalized collision energy of 40%). Cycle time was 0.2 msec, which in Astral was equivalent to approximately top-35 DDA-MS/MS. Electrospray ionization settings: spray voltage 3500 v (+), sheath gas 60 arbitrary units, axillary gas 20 arbitrary units, sweep gas 1 arbitrary unit, ion transfer tube temperature 350 °C, vaporizer temperature 400 °C, RF lens 50%. Plasma samples were extracted by a biphasic solution of MTBE/methanol/water as previously published^41^, and aliquots were dried and resuspended in 100 μL of ACN:water (80:20) containing 30 isotope-labeled internal standards. 3 μl was injected. Pooled quality control samples, including reference material NIST SRM1950 plasma and blank quality controls, were analyzed to assess quantitative robustness and selectivity. Feature detection and alignment were performed using MS-DIAL (version 4.9.2). Compound annotations were performed using combined repositories of NIST23, Massbank.us, and GNPS libraries. Candidate spectra for identity search using entropy similarity and *Denoising Search* were restricted to a precursor ion mass tolerance of 10 mDa. Compound superclass information was assigned using the ClassyFire algorithm^42^.

## Data Availability

NIST Tandem Mass Spectral Library, 2023 release (NIST23) spectra are commercial available and can be purchased from multiple vendors. MassBank of North America database (Massbank.us) spectra can be freely downloaded from Massbank.us (https://massbank.us/). The metabolome dataset of Alzheimer’s Disease samples and the experimental data from the chemical dilution series can be requested from the authors. Source data are provided with this paper.
